# Genome-Wide Association Study of Tacrolimus Pharmacokinetics Identifies Novel Single Nucleotide Polymorphisms in the Convalescence and Stabilization Periods of Post-transplant Liver Function

**DOI:** 10.3389/fgene.2019.00528

**Published:** 2019-05-31

**Authors:** Yuan Liu, Chengdong Zhang, Lei Li, Baochi Ou, Liyun Yuan, Tao Zhang, Junwei Fan, Zhihai Peng

**Affiliations:** ^1^Department of Hepatobiliary Pancreatic Surgery, Shanghai General Hospital, School of Medicine, Shanghai Jiao Tong University, Shanghai, China; ^2^State Key Laboratory of Genetic Engineering, School of Life Sciences, Fudan University, Shanghai, China; ^3^Key Lab of Computational Biology, CAS-MPG Partner Institute for Computational Biology, Shanghai Institutes for Biological Sciences, Chinese Academy of Sciences, Shanghai, China; ^4^Department of Organ Transplant, The Second Affiliated Hospital to Guangzhou Medical University, Guangzhou, China

**Keywords:** GWAS, CYP3A5, exome, tacrolimus, FAM26F

## Abstract

After liver transplantation, the liver function of a patient is gradually restored over a period of time that can be divided into a convalescence period (CP) and a stabilizing period (SP). The plasma concentration of tacrolimus, an immunosuppressant commonly used to prevent organ rejection, varies as a result of variations in its metabolism. The effects of genetic and clinical factors on the plasma concentration of tacrolimus appear to differ in the CP and SP. To establish a model explaining the variation in tacrolimus trough concentration between individuals in the CP and SP, we conducted a retrospective, single-center, discovery study of 115 pairs of patients (115 donors and 115 matched recipients) who had undergone liver transplantation. Donors and recipients were genotyped by a genome-wide association study (GWAS) using an exome chip. Novel exons were identified that influenced tacrolimus trough concentrations and were verified with bootstrap analysis. In donors, two single-nucleotide polymorphisms showed an effect on the CP (rs1927321, rs1057192) and four showed an effect on the SP (rs776746, rs2667662, rs7980521, rs4903096); in recipients, two single-nucleotide polymorphisms showed an effect in the SP (rs7828796, rs776746). Genetic factors played a crucial role in tacrolimus metabolism, accounting for 44.8% in the SP, which was higher than previously reported. In addition, we found that CYP3A5, which is known to affect the metabolism of tacrolimus, only influenced tacrolimus pharmacokinetics in the SP.

## Introduction

Tacrolimus is one of the most common and potent immunosuppressive drugs used to lower the risk of acute organ rejection in patients through the suppression of the cytotoxic activity of the T lymphocytes involved in acute rejection; however, it has a narrow therapeutic window (Liu et al., 1991; Kelly et al., 2004; Wu et al., 2018). The post-operation blood concentration of tacrolimus should be strictly monitored in accordance with the recommended time-dependent target trough concentrations, as subtherapeutic doses of tacrolimus are associated with acute rejection and graft loss (Rehman et al., 2014) and overdosing of tacrolimus produces adverse side effects, such as nephrotoxicity and new-onset diabetes (Kershner and Fitzsimmons, 1996; Shi et al., 2013; Ling et al., 2017). The metabolism of tacrolimus, which plays an essential role in its blood concentration, is largely dependent on the cytochrome P450 3A (CYP3A) subfamily, especially CYP3A5 (Mourad et al., 2005). As the genetic polymorphisms of CYP3A5 affect its expression and activity, the effects of CYP3A5 polymorphisms on the metabolism of tacrolimus have been studied thoroughly over the last few decades to provide an understanding of the variance in tacrolimus blood concentrations among patients (Alvarez-Elias et al., 2016; Deininger et al., 2016). Patients with the CYP3A5^∗^3/^∗^3 genotype (non-expressors) receiving orthotopic liver transplantation (OLT) or renal transplantation require a lower tacrolimus dose to reach similar trough levels compared with those with the CYP3A5^∗^1 allele (expressors) (MacPhee et al., 2005; Renders et al., 2007; Argudo et al., 2015; Kato et al., 2016). In addition to CYP3A5, single nucleotide polymorphisms (SNPs) of other genotypes have been implicated in tacrolimus metabolism, including CYP3A4 and recipient ABCB1 (adenosine triphosphate-binding cassette sub-family B member 1) (Shi et al., 2013; Deininger et al., 2016; Debette-Gratien et al., 2016), although no consensus has been reached. In the present study, considering that multiple unknown genes may be involved in tacrolimus metabolism, we performed a genome-wide association study (GWAS) to evaluate more than 240,000 exonic variants in 115 donors and 115 matched recipients with the associated tacrolimus trough concentrations using an exome chip, and established valid models to account for the variation in tacrolimus concentrations.

## Materials and Methods

### Study Subjects

In this study, 115 donors and 115 matched recipients receiving an OLT between July 2015 and March 2017 at the First People’s Hospital, affiliated to Shanghai Jiao Tong University School of Medicine, and registered in the China Liver Transplant Registry (CLTR) database, were enrolled. All donors were from donation after brain death. This study was registered in ClinicalTrials.gov and the identification number is NCT02752529. All recipients followed the same post-operation treatment protocol: oral tacrolimus, 0.1 mg/kg/day; steroids, 0.8–1.0 mg/kg/day; mycophenolate, 1.5 g/day; and lamivudine combined with low-dose intramuscular hepatitis B immunoglobulin therapy for anti-viral treatment in recipients with hepatitis B virus-related liver disease. If patients had an acute rejection reaction or renal function insufficiency, we increased or decreased the dose of anti-rejection drugs, as appropriate. The enrollment criteria were: (i) adult patients (≥18 years of age), (ii) patients that received a tacrolimus-based immunosuppressive regime. The exclusion criteria were: (i) multiorgan transplant patients; (ii) follow-up time less than 1 month; (iii) incomplete patient data. All recipients had associated follow-up information, including tacrolimus trough concentrations. Patients with pre-operative abnormal renal function were excluded. Informed consent was obtained from all subjects or their direct relatives. The study was approved by the Institutional Review Board and conducted strictly in accordance with the guidelines of the Ethics Committee of the Shanghai First Hospital affiliated with Shanghai Jiao Tong University. The methods were conducted in accordance with the Declaration of Helsinki and its later amendments.

### Data Collection

The individual medical records of each enrolled subject were reviewed and recorded at the time of inclusion in the study. The age, gender, body mass index (BMI), blood type, and HLA mismatch of all included subjects were collected. The clinical laboratory index of recipients before and after operation, including glutamic-pyruvic transaminase (ALT), total bilirubin (TB), creatinine (Cr), hemoglobin (Hb), and direct bilirubin (DB), was measured uniformly in the clinical laboratory of the Shanghai General Hospital, affiliated to Shanghai Jiao Tong University. Tacrolimus trough concentrations were measured before morning administration in the first 4 weeks post-operation. Natural logarithms of tacrolimus dose-normalized tacrolimus trough concentration [ln (TAC C_0_/D ratio)] (nanograms per milliliter per total daily dose in milligrams, ng/mL/mg) were calculated before applying statistical analysis.

### Genotyping and Genome-Wide Association Study (GWAS)

Liver tissue (20–50 mg) was extracted from each donor and recipient, and tacrolimus trough concentration was measured in the whole blood by using the Pro-TracTMII tacrolimus ELISA kit (Diasorin, Stillwater, MN, United States) with a microparticle enzyme immunoassay (ELx 800NB analyzer, BioTek, Winooski, VT, United States). The genomic DNA was collected by using the AllPrep DNA Mini Kit (QIAGEN, Hilden, Germany). DNA concentration was quantified by using a NanoDrop ND2000 spectrophotometer (NanoDrop Technologies, Wilmington, DE, United States). All DNA samples were amplified in two separate multiplex PCR assays. PCR products were cleaned up using AMPure XP Beads (Beckman Coulter, Pasadena, CA, United States) (Lang et al., 2017). Genotypes of all samples were determined by Infinium Human Exome-12 v1.2 BeasChip accessing > 240,000 exonic variants. The CYP3A5 genotype was selected from the Drug Metabolizing Enzymes and Transporters (DMET) chip (Liu et al., 2017).

### Statistical Analysis

Quantitative variables were expressed as the mean ± standard deviation (SD) or the median and interquartile range (IQR). Categorical variables were presented as values and compared by using Pearson’s χ^2^ test. The minor allele frequency (MAF) was set as 0.1. The Hardy–Weinberg Equilibrium (HWE) test was performed using an appropriate χ^2^ test. Pairwise *R*^2^ and *D*-values for linkage disequilibrium were calculated by using SHEsis software^1^. SNPs of raw *P*-value with significance were then analyzed according to intersections between 4 weeks of post-operation data collected separately from donors and recipients. SNPs in these intersections were further verified through univariate and multivariate regression analysis, together with clinical baseline characteristics. The significant variables, as determined by multivariate regression analysis, were used to establish a model that could account for the ln(TAC C_0_/D ratio) observed in the convalescence period (CP) and the stabilizing period (SP). Genotype data analysis and quality control were performed by using PLINK software (Purcell et al., 2007)^2^. SPSS version 22.0 (SPSS Inc., Chicago, IL, United States) and GraphPad Prism 7 (GraphPad Prism Software Inc., San Diego, CA, United States) were used to perform statistical analysis. Manhattan plots and Venn diagrams were generated by using R Studio^3^. A two-tailed *P*-value of < 0.05 was deemed significant.

## Results

### Baseline Characteristics

In the study, 115 donors (114 male and 1 female) and 115 recipients (96 male and 19 female) were enrolled in the study. The ages and BMI of donors were not available. There was no significant difference in gender between donors and recipients (*P* = 1.0). The MELD (model for end stage of liver disease) score of recipients was 11.4 ± 5.9. The clinical laboratory indices of recipients pre- and post-operation are detailed in Table 1. The median value of dose-normalized tacrolimus trough concentrations was 5.3 ng/mL/mg (IQR: 4.8–5.8 ng/mL/mg) in Weeks 1–2, and 4.7 ng/mL/mg (IQR: 4.3–5.3 ng/mL/mg) in Weeks 3–4.

**Table 1:** Characteristic of patients.

	Recipient (*n* = 115)
Age, years (mean ± SD)	47.3 ± 9.0
Gender, male/female, n	96/19
BMI, kg/m2, (mean ± SD)	23.1 ± 5.0
MELD score (mean ± SD)	11.4 ± 5.9
ALT.pre, U/l, median (IQR)	18.0 (12.0–39.0)
TB.pre, μmol/L, median (IQR)	23.0 (13.0–48.0)
Cr.pre, mg/dl, median (IQR)	60.0 (47.0–70.0)
Hb.pre, g/L, median (IQR)	107.0 (81.0–134.0)
ALT.post, U/l, median (IQR)	86.6 (62.8–131.8)
TB.post, μmol/L, median (IQR)	36.5 (21.7–63.0)
Cr.post, μmol/L, median (IQR)	57.4 (51.3–74.8)
Hb.post, g/L, median (IQR)	97.9 (89.3–107.7)
Tacrolimus dose normalized tacrolimus trough in weeks 1–2, ng/mL/mg, median (IQR)	5.3 (4.8–5.8)
Tacrolimus dose normalized tacrolimus trough in weeks 3–4, ng/mL/mg, median (IQR)	4.7 (4.3–5.3)

*#, missing; BMI, body mass index; ALT.pre, glutamic-pyruvic transaminase detected before transplant; TB.pre, total bilirubin detected before transplant; Cr.pre, creatinine detected before transplant; Hb.pre, hemoglobin detected before transplant; ALT.post, glutamic-pyruvic transaminase detected after transplant; TB.post, total bilirubin detected after transplant; Cr.post, creatinine detected after transplant; Hb.post, hemoglobin detected after transplant; IQR, interquartile range.*

### Genome-Wide Association Study

**FIGURE 1:**
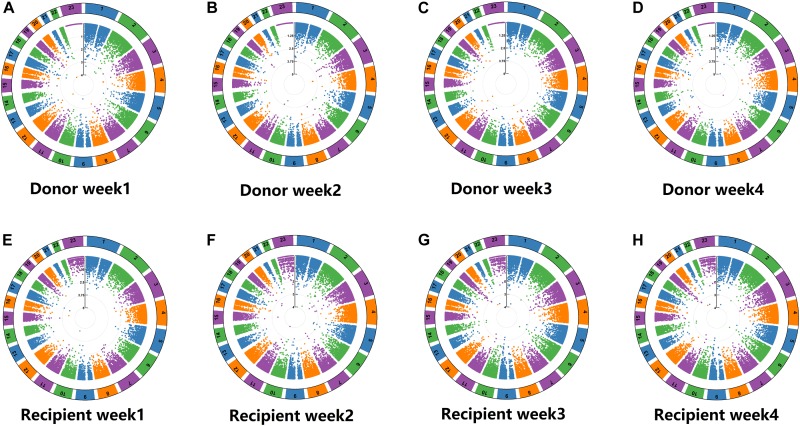
Manhattan plots of single-nucleotide polymorphisms (SNPs) associated with ln(TAC C_0_/D ratio) in donors and recipients respectively. Total > 240,000 markers were analyzed toward natural log of dose-normalized tacrolimus trough concentrations between donors and recipients in the first 4 weeks with adjustment of age, donor gender, gender and BMI. All SNPs were shown in order from chromosome 1 to 23 (not including Y chromosome). **(A)** Manhattan plots of single-nucleotide polymorphisms (SNPs) associated with ln(TAC C_0_/D ratio) in donors in week 1. **(B)** Manhattan plots of single-nucleotide polymorphisms (SNPs) associated with ln(TAC C_0_/D ratio) in donors in week 2. **(C)** Manhattan plots of single-nucleotide polymorphisms (SNPs) associated with ln(TAC C_0_/D ratio) in donors in week 3. **(D)** Manhattan plots of single-nucleotide polymorphisms (SNPs) associated with ln(TAC C_0_/D ratio) in donors in week 4. **(E)** Manhattan plots of single-nucleotide polymorphisms (SNPs) associated with ln(TAC C_0_/D ratio) in recipients in week 1. **(F)** Manhattan plots of single-nucleotide polymorphisms (SNPs) associated with ln(TAC C_0_/D ratio) in recipients in week 2. **(G)** Manhattan plots of single-nucleotide polymorphisms (SNPs) associated with ln(TAC C_0_/D ratio) in recipients in week 3. **(H)** Manhattan plots of single-nucleotide polymorphisms (SNPs) associated with ln(TAC C_0_/D ratio) in recipients in week 4.

GWAS was performed using > 240,000 markers on the 115 patient pairs (115 donors and 115 recipients), evaluating the association between SNPs and the natural log of tacrolimus concentration/dose ratios between donors and recipients in the first 4 weeks after OLT, adjusted for age, donor gender, recipient gender, and BMI. All SNPs were shown in order from chromosome 1 to 23 (excluding the Y chromosome). P-values were calculated for all SNPs and were shown in a Manhattan plot (Figure 1). The total number of significant correlative SNPs in donors was 105 in Week 1, 103 in Week 2, 123 in Week 3, and 113 in Week 4; in recipients, the number was 138 in Week 1, 132 in Week 2, 138 in Week 3, and 117 in Week 4 (Supplementary Table S1).

### Analysis of Differences in Liver Function in Convalescence and Stabilizing Periods

**FIGURE 2:**
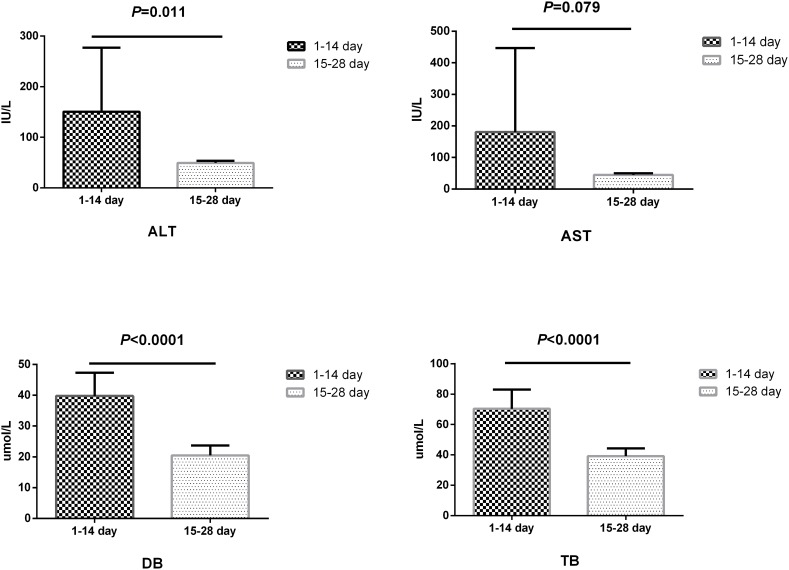
Differences of convalescence phase and stabilizing phase of liver function. ALT, glutamic-pyruvic transaminase; AST, glutamic oxalacetic transaminase; TB, total bilirubin; DB, direct bilirubin. *P* < 0.05 was considered significant.

The liver function of recipients was followed up over the first 4 weeks post-operation as represented by four indicators: alanine aminotransferase (ALT), aspartate aminotransferase (AST), direct bilirubin (DB), and total bilirubin (TB). All four indicators were higher in the first 2 weeks (the convalescence period of liver function; CP) than in the latter 2 weeks (the stabilizing period of liver function; SP). Significant differences between the two phases were observed in ALT (*P* = 0.011), DB (*P* < 0.0001), and TB (P < 0.0001) levels, and the difference in AST almost reached statistical significance (*P* = 0.078) (Figure 2).

### Correlative SNPs Between CP and SP in Donors and Recipients

**FIGURE 3:**
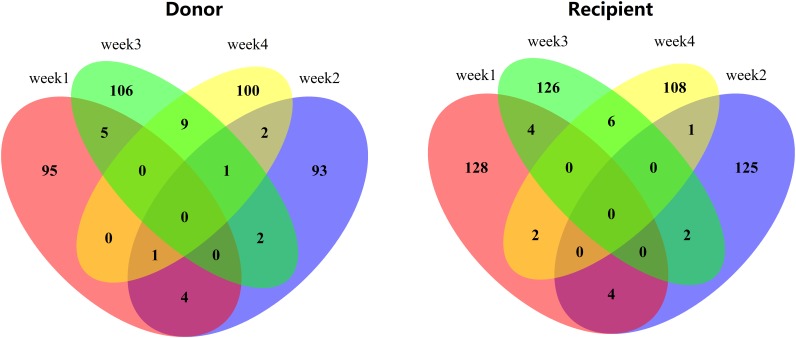
Venn diagram showed the correlation between week1 and week2 (convalescence phase), as well as week3 and week4(stabilizing phase) in donors and recipients respectively. The numbers meant the number of intersections between or among weeks. 0 meant no intersection.

A Venn diagram of the correlative SNPs with statistical significance in the GWAS of the CP and SP is shown in Figure 3. The number of intersections in donors was 5 in the CP (intersection between the red and blue ellipses) and 10 in the SP (intersection between the green and yellow). In recipients, the numbers of intersections in the CP and SP were 4 and 6, respectively. Specific genotypes are detailed in Tables 2, 3.

**Table 2:** Univariate and multivariate regression analysis in convalescence phase.

					Univariate				Multivariate			
Origin	Rs	Gene	Chr	Position	β	*P*-value	Bootstrap β	Bootstrap *P*-value	β	*P*-value	Bootstrap β	Bootstrap*P*-value
Donor	rs776746	CYP3A5	7	99672916	0.286	**0.008**	0.286	**0.008**	0.194	0.058	47.733	0.062
Donor	rs12677741	PXDNL	8	52529097	−0.011	0.909	−0.011	0.904				
Donor	rs1058029	TMX4	20	7961715	−0.208	0.412	−0.208	0.464				
Donor	rs1927321		9	1.21E+08	0.310	**0.001**	0.310	**0.007**	0.233	**0.008**	0.233	**0.015**
Donor	rs1827293	NBPF3	1	21795388	−0.158	0.078	−0.158	0.073				
Donor	rs1057192	FAM26F	6	1.17E+08	−0.345	**0.000**	−0.345	**0.001**	−0.289	**0.001**	−0.289	**0.001**
Recipient	rs11605576	SLC22A20	11	64981522	0.013	0.912	0.013	0.922				
Recipient	rs11605632	SLC22A20	11	64981587	0.013	0.912	0.013	0.931				
Recipient	rs12420456	SLC22A20	11	64981837	0.013	0.912	0.013	0.923				
Recipient	rs3826736	C19orf45	19	7571030	0.263	**0.012**	0.263	**0.019**	0.162	0.101	0.162	0.067
Recipient	rs776746	CYP3A5	7	99672916	0.312	**0.004**	0.312	**0.017**	0.204	0.044	0.204	0.090
Recipient	Age				0.000	0.968	0.000	0.979				
Recipient	Gender				−0.069	0.704	−0.069	0.673				
Donor	Gender				−0.040	0.956	−0.040	0.615				
Recipient	BMI				0.027	0.295	0.027	0.295				
Recipient	MELD score				0.011	0.323	0.011	0.277				
Recipient	ALT.pre				0.002	0.084	0.002	**0.030**	0.000	0.773	0.000	0.732
Recipient	TB.pre				0.001	0.125	0.001	0.278				
Recipient	Cr.pre				0.004	0.069	0.004	**0.045**	0.004	**0.022**	0.004	**0.026**
Recipient	Hb.pre				0.001	0.707	0.001	0.663				

*BMI, body mass index; ALT.pre, glutamic-pyruvic transaminase detected before transplant; TB.pre, total bilirubin detected before transplant; Cr.pre, creatinine detected before transplant; Hb.pre, hemoglobin detected before transplant. Bold values indicate the significant level at P < 0.05.*

**Table 3:** Univariate and multivariate regression analysis in stabilizing phase.

					Univariate				Multivariate			
Origin	rs	Gene	Chr	Position	β	*P*-value	Bootstrap β	Bootstrap *P*-value	β	*P*-value	Bootstrap β	Bootstrap *P*-value
Donor	rs776746	CYP3A5	7	99672916	0.349	**0.001**	0.349	**0.001**	0.308	**0.000**	0.336	**0.014**
Donor	rs6678469	KAZN	1	14281451	0.237	**0.008**	0.237	**0.006**	0.045	0.540	0.043	0.558
Donor	rs2667662	TELO2	16	1551082	0.282	**0.002**	0.282	**0.001**	0.221	**0.003**	0.218	**0.014**
Donor	rs7980521	ESYT1	12	56535251	−0.295	**0.002**	−0.295	**0.005**	−0.283	**0.000**	−0.283	**0.014**
Donor	rs4401444	LOC101928987	4	82620131	−0.209	**0.046**	−0.209	**0.048**	−0.099	0.162	−0.099	0.156
Donor	rs4903096	–	14	40329432	−0.318	**0.001**	−0.318	**0.001**	−0.269	**0.000**	−0.269	**0.007**
Donor	rs8916	EIF4B	12	53433486	0.431	**0.000**	0.431	**0.002**	0.113	0.142	0.113	0.156
Donor	rs6719889	LOC101927261	2	58237076	−0.141	0.230	−0.141	0.102				
Donor	rs2302310	QRFPR	4	122250734	−0.002	0.988	−0.002	0.988				
Donor	rs9778	ESD	13	47354101	−0.164	0.078	−0.164	0.085				
Donor	rs2276231	LOC105372795	21	38437951	0.091	0.706	0.091	0.661				
Recipient	rs17263971	SYNPO2	4	1.2E+08	0.412	**0.004**	0.412	**0.005**	0.138	0.065	0.138	0.095
Recipient	rs7828796	–	8	84280532	0.282	**0.002**	0.282	**0.006**	0.155	**0.030**	0.155	**0.048**
Recipient	rs13036385	BPIFB4	20	31671209	−0.306	**0.021**	−0.306	**0.031**	−0.033	0.655	−0.033	0.687
Recipient	rs4339026	BPIFB4	20	31671599	0.306	**0.021**	0.306	**0.040**	0.204	**0.004**		#
Recipient	rs7643736	–	3	3767186	0.028	0.762	0.028	0.767				
Recipient	rs2293925	TOP1MT	8	1.44E+08	−0.108	0.257	−0.108	0.297				
Recipient	rs776746	CYP3A5	7	99672916	0.286	**0.009**	0.286	**0.005**	0.238	0.004	0.238	**0.007**
Recipient	Age				−0.005	0.484	−0.005	0.543				
Recipient	Gender				−0.135	0.456	−0.135	0.459				
Donor	Gender				0.127	0.861	0.127	0.132				
Recipient	BMI				−0.005	0.484	−0.005	0.552				
Recipient	MELD score				0.011	0.349	0.011	0.265				
Recipient	ALT.post				0.001	0.304	0.001	0.316				
Recipient	TB.post				0.004	0.006	0.004	0.022				
Recipient	Cr.post				5.117E-5	0.980	5.117E-5	0.972				
Recipient	Hb.post				−0.008	0.098	−0.008	0.179				

*BMI, body mass index; ALT.post, glutamic-pyruvic transaminase detected after transplant; TB.post, total bilirubin detected after transplant; Cr.post, creatinine detected after transplant; Hb.post, hemoglobin detected after transplant. Bold values indicate the significant level at P < 0.05.*

### Analysis of Correlative SNPs and Clinical Variables in Donors and Recipients and Their Relationship With ln(TAC C_0_/D Ratio) in the CP and SP

The association of the correlative SNPs of donors and recipients with ln(TAC C_0_/D ratio) was explored by univariate and multivariate analyses in the CP and SP. Clinical variables were simultaneously applied to the regression analysis. The significant variables, as identified by univariate analysis, were then applied to multivariate analysis. The results of the multivariate analysis revealed that there were three significant variables linked to tacrolimus metabolism in CP (Table 2): donor rs1927321 (*P* = 0.008, bootstrap *P* = 0.015), donor rs1057192 (*P* = 0.001, bootstrap *P* = 0.001), and preoperative creatine (*P* = 0.022, bootstrap *P* = 0.026). In SP (Table 3), six significant variables were identified, namely: donor rs776746 (*P* = 0.000, bootstrap *P* = 0.014), donor rs2667662 (*P* = 0.003, bootstrap *P* = 0.014), donor rs7980521 (*P* = 0.000, bootstrap *P* = 0.014), donor rs4903096 (*P* = 0.000, bootstrap *P* = 0.007), recipient rs7828796 (*P* = 0.030, bootstrap *P* = 0.048), and recipient rs776746 (*P* = 0.004, bootstrap *P* = 0.007).

### Model of Variation in Tacrolimus Trough Concentration in the CP and SP

A model was constructed by using significant SNPs and clinical variables to explain variation in tacrolimus trough concentrations in the CP and SP by linear regression respectively (Table 4). In CP, donor rs1927321 was responsible for 8.9% variance; donor rs1057192 was responsible for 11.1% variance, and Cr.pre was responsible for 2.9% variance. In SP, donor rs776746, donor rs2667662, donor rs7980521, donor rs4903096, recipient rs7828796, recipient rs776746 were responsible for 10.3, 8.2, 8.5, 10.3, 8.1, and 6.0% variance, respectively. Overall, the proportion of variation explained by this model was 22.0% in CP (adjusted *R*^2^ = 19.9%) and 47.8% in SP (adjusted *R*^2^ = 44.8%).

## Discussion

Liver transplantation is a major surgical operation, and the liver and gastrointestinal tract of post-operative recipients needs a recovery period to achieve a return to normal physiological functions. Liver function did not recover in the early period after liver transplant (CP), as at that time, the donor liver could not completely metabolize tacrolimus and the genetic function could not be observed. After the donor liver function recovered (in the SP), Tacrolimus could be metabolized completely, which indicated the donors’ genetic function. During these two different periods, the genetic and clinical factors affecting tacrolimus metabolism may change. We used an exon microarray in addition to measurements of pre- and post-operative clinical indicators to explore the factors influencing tacrolimus metabolism in the different periods of recovery after liver transplantation and used this information to build appropriate models. In addition, we selected the genotype of CYP3A5, known to have a significant effect on tacrolimus metabolism, from the DMET chip. Our study identified two novel SNPs (rs1927321, rs1057192) in donors in the CP, four novel SNPs (rs776746, rs2667662, rs7980521, rs4903096) in donors in the CP, and two novel SNPs (rs7828796, rs776746) in recipients in the SP.

**Table 4:** Tacrolimus variance in natural log–transformed dose-normalized tacrolimus troughs explained by a model in recovery phase and stabilizing phase respectively.

Model	Tacrolimus variation explained by model, %	
	*R*^2^	Adjusted *R*^2^
**Recovery phase**		
D rs1927321	8.9	8.1
D rs1057192	11.1	10.3
Cr.pre	2.9	2.1
Cr.pre+ D rs1927321 and D rs1057192	22.0	19.9
**Stabilizing phase**		
D rs776746	10.3	9.5
D rs2667662	8.2	7.3
D rs7980521	8.5	7.7
D rs4903096	10.3	9.5
R rs7828796	8.1	7.3
R rs776746	6.0	5.1
D rs776746, D rs2667662, D rs7980521, D rs4903096, R rs7828796, and R rs776746	47.8	44.8

*D meant single nucleotide polymorphisms (SNPs) originated from a donor, R meant single nucleotide polymorphisms (SNPs) originated from the recipient; Cr.pre, creatinine detected before transplant.*

The CYP3A5 genotype is a well-known factor in tacrolimus metabolism *in vitro* and *in vivo*, and is currently the most reliable predictor of an individual’s tacrolimus dose requirement (Kamdem et al., 2005; Birdwell et al., 2015). As the metabolism of tacrolimus by CYP3A5 occurs mainly in the liver and intestine, CYP3A5 genotypes are relevant in both the donor liver allografts and the recipient native intestines (Masuda and Inui, 2006). Ji et al. (2012) reported that the donor CYP3A5 genotype had a minimal influence on tacrolimus metabolism in CYP3A5 non-expressor recipients during the first month, and that the effect gradually changed over time. In our study, we found that CYP3A5 polymorphisms did not correlate with variations in the tacrolimus trough concentration in either donors or recipients and was thus excluded from the model in the CP. During the SP, the association became more prominent, which indicated that the CYP3A5 polymorphism of donors and recipients did exert a short-term influence on tacrolimus metabolism in the short term, and that this influence amplified over time and may be even more remarkable in the long term.

Our findings of several novel SNPs as independent relevant factors affecting tacrolimus metabolism have not been previously described; these SNPs and their effects may provide new insights into the direction of future research. Among these SNPs, rs266762 is located in TELO2 (telomere maintenance 2), a gene involved in the maintenance of telomere length (You et al., 2016). The TELO2 protein can interact with phosphatidylinositol 3-kinase-related protein kinases and is a component of mTOR (mammalian target of rapamycin) (Fernandez-Saiz et al., 2013). mTOR can protect the liver from ischemia- or reperfusion-induced injury through the NF-kB pathway. Zhang et al. (2017) reported that NF-kB suppressed the expression of PXR (pregnane X receptor)-mediated CYP3A5 gene. Hence, TELO2 rs26672 might affect tacrolimus pharmacokinetics through the mTOR/NF-kB/CYP3A5 pathway. ESYT1 (Extended synaptotagmin 1) rs7980521 is located on chromosome 12 with major allele G. ESYT1 is an endoplasmic reticulum protein that binds to the plasma membrane and transports lipids (Bian et al., 2018). We supposed that ESYT1 rs7980521 could affect tacrolimus metabolism through lipid transportation because tacrolimus is a fat-soluble drug. Rs1057192 is located in FAM26F (gene family with sequence similarity 26, member F), a recently identified gene reported to be involved in manifold immune responses (Malik et al., 2017). Interestingly, FAM26F has been found in several gene signatures associated with oxidative stress and inflammation in the study of liver ischemia-reperfusion injury in liver transplantation (Defamie et al., 2008). In our final model, SNPs within FAM26F accounted for 11.2%, the largest share, of the tacrolimus trough variation in the CP. Therefore, FAM26F may play an intriguing role in liver transplantation and further investigation into the associated genotypes may yield meaningful results.

The influence of other covariates (such as demographics, clinical laboratory indices, disease-related factors, and cotreatments) on tacrolimus metabolism has been extensively investigated (Venkataramanan et al., 1995; Campagne et al., 2018). In our study, we included some frequently reviewed covariates, including age, sex, BMI, MELD score, hematocrit, liver function (levels of AST, ALT, DB, and TB), and serum creatinine. Among all the covariates we studied, pre-operation serum creatinine concentration of recipients in the CP was the only variable that showed statistical significance. In keeping with our findings, serum creatinine was previously shown to be relevant in tacrolimus metabolism (Wang et al., 2017), whereas the other baseline characteristics we studied have been reported to have no effect. Interestingly, serum creatinine only influenced tacrolimus metabolism during CP, showing that, to a certain extent, the major factors affecting tacrolimus metabolism were genetic. The proportion of variation in metabolism explained by our model was 22.0% in the CP and 47.8% in the SP, which is, to the best of our knowledge, the highest variation reached (Oetting et al., 2016). This suggested that the effects of genetic factors may be weak or may not occur in the CP, but become stronger in the SP.

There are, unfortunately, several limitations to our study. First, donor demographic data were limited. Fortunately, the quality of donor livers was strictly assessed to satisfy transplantation standards. Under these conditions, the genetic factors were critical. In addition, we did not perform functional testing of the genetic loci as the focus of this article was the identification of key SNPs for further study.

## Conclusion

We found that SNPs play a non-negligible role in tacrolimus metabolism, and that their role changed over time with the recovery of liver function. The novel SNPs we identified with effects on tacrolimus metabolism could deliver promising new insights into the study of the relationship between patient genotypes and tacrolimus metabolism. The two-phase models we developed based on genetic polymorphism and clinical covariates accounted for a significant portion of the inter-individual variation in tacrolimus trough concentrations and may contribute to the optimization of individual tacrolimus dosing.

## Data Availability

The datasets generated for this study can be found in the GEO https://www.ncbi.nlm.nih.gov/geo/query/acc.cgi?acc=GSE130068.

## Ethics Statement

Informed consent was agreed and signed by all subjects or their direct relatives. The study was approved by the Institutional Review Board, and carried out strictly abide by the guidelines of the Ethics Committee of the Shanghai First Hospital Affiliated Shanghai Jiao Tong University. The methods were carried out in accordance with the Declaration of Helsinki and its later amendments.

## Author Contributions

YL and CZ performed all studies. YL and LL drafted the manuscript. YL, BO, and TZ collected the data. YL and LY performed the statistical analysis. ZP and JF participated in the experimental design. All authors read and approved the final manuscript.

## Conflict of Interest Statement

The authors declare that the research was conducted in the absence of any commercial or financial relationships that could be construed as a potential conflict of interest.

## Footnotes

1 http://analysis.bio-x.cn/myAnalysis.php

2 http://www.cog-genomics.org/plink2

3 https://www.rstudio.com/products/rstudio

## Supplementary Material

TABLE S1 SNPs influencing Tacrolimus pharmacikonetics in CP and SP respectively.
